# A case of urticarial vasculitis associated with atezolizumab

**DOI:** 10.1016/j.jdcr.2023.09.042

**Published:** 2023-11-20

**Authors:** Jade N. Young, Ryan Rivera-Oyola, Dina Poplausky, Yamato Suemitsu, Randie H. Kim, Deborah Doroshow, Nicholas Gulati

**Affiliations:** a Department of Dermatology, Icahn School of Medicine at Mount Sinai, New York, New York; b Department of Pathology, Molecular and Cell-Based Medicine, Icahn School of Medicine at Mount Sinai, New York, New York; c Division of Hematology and Medical Oncology, Department of Medicine, Tisch Cancer Institute, Icahn School of Medicine at Mount Sinai, New York, New York

**Keywords:** atezolizumab, cirAE, cutaneous immune-related adverse event, immune checkpoint inhibitor, immune-related adverse event, irAE, urticarial vasculitis, vasculitis

## Introduction

Immune checkpoint inhibitors (ICIs) have revolutionized the treatment of various cancers. The nonspecific immune activation induced by ICIs can lead to immune-related adverse events (irAEs).^1^ Cutaneous irAEs (cirAEs) are the most common, encompassing a spectrum of skin toxicities that mimic inflammatory skin conditions.^2^ ICIs are also associated with rheumatologic irAEs presenting with cutaneous manifestations, including systemic lupus erythematosus, dermatomyositis, and vasculitis, with large vessel vasculitis as the most commonly associated vasculitis.^2^, ^3^, ^4^ As the utilization of ICIs becomes increasingly widespread, it is expected that additional cutaneous toxicities will be associated with these agents.

Urticarial vasculitis is a rare form of vasculitis characterized by chronic or recurrent, typically painful urticarial plaques persisting >24 hours.^5^ In this manuscript, we present a case of urticarial vasculitis in a patient undergoing ICI treatment with the programmed death-ligand 1 (PD-L1) inhibitor atezolizumab.

## Case report

A 70-year-old female with metastatic small cell lung cancer presented with a 1-month history of mildly tender, pruritic raised welts. Two to 3 new lesions appeared daily, never in the same location. The lesions lasted 2 to 3 days before regressing. The patient reported a prodromal sensation of itch preceding the development of each lesion. She denied associated systemic symptoms such as joint pain. The initial lesion emerged 19 days after commencing cancer therapy with atezolizumab (cycle 1) and persisted through cycle 2, with infusions administered every 3 weeks. Pantoprazole was the only concomitant medication, initiated 4 months before rash onset. Treatment with oral antihistamines and clobetasol 0.05% ointment was attempted but provided minimal relief.

**Fig 1:**
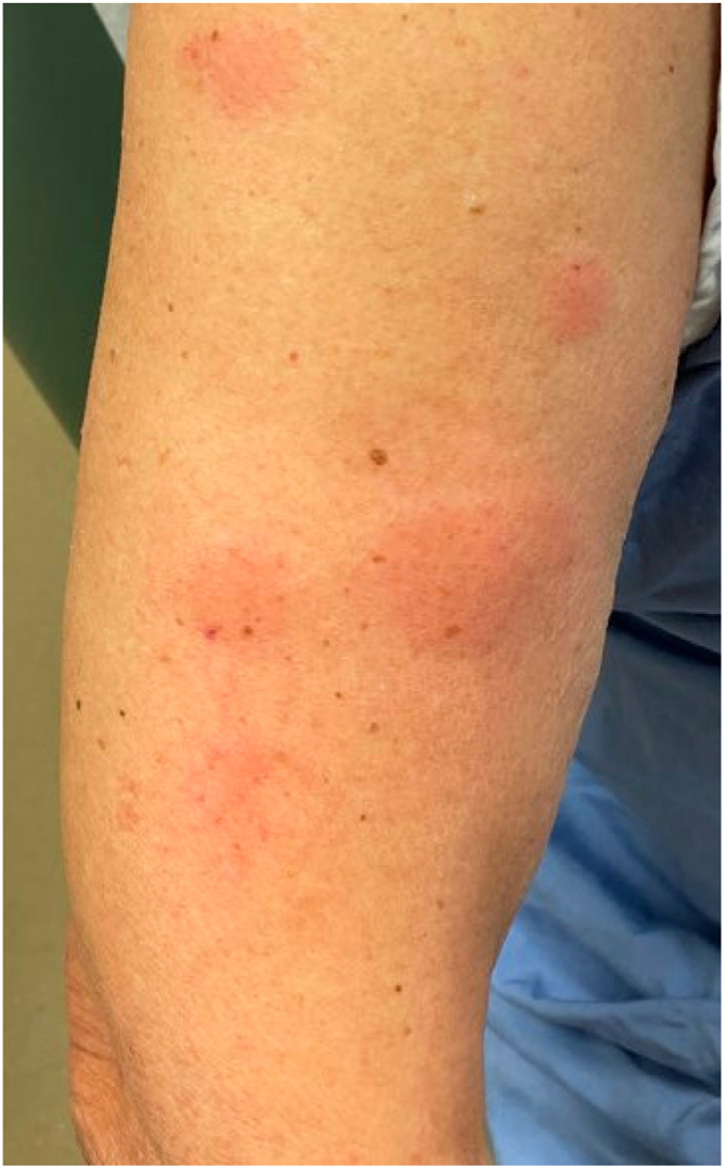
Faintly erythematous, edematous tender plaques on the right arm lasting >24 hours consistent with urticarial vasculitis.

**Fig 2:**
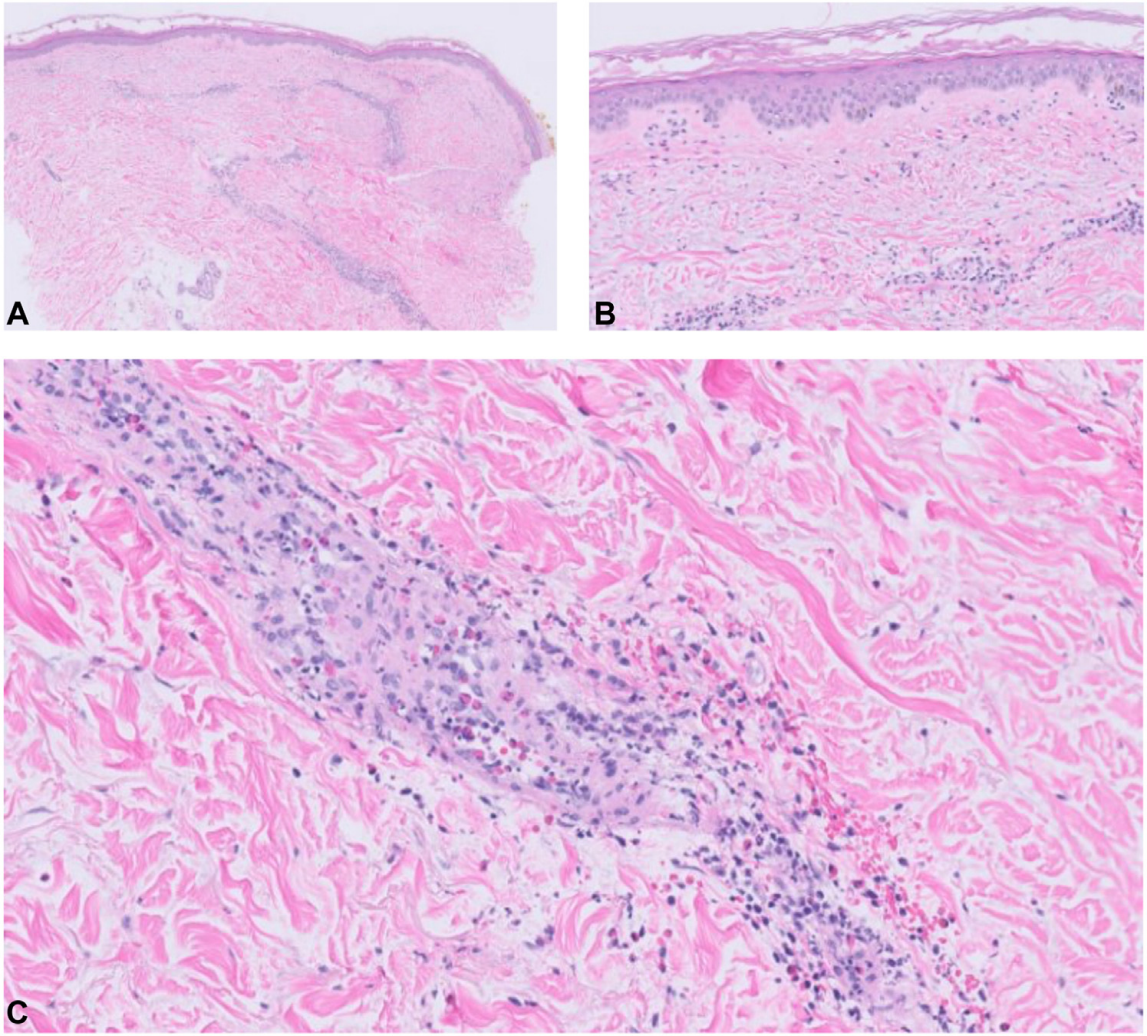
Histopathology demonstrating sparse perivascular dermatitis (**A**, ×50, low power), neutrophilic and eosinophilic infiltrate (**B**, ×200 interstitial), neutrophils within the blood vessel walls with leukocytoclasia, extravasated erythrocytes, and hints of fibrin deposition within the vessel walls (**C**, ×200 perivascular).

On physical examination, faintly erythematous, edematous plaques were observed on the bilateral arms, legs, face, scalp, and buttocks (Fig 1). Histopathologic examination revealed a superficial-to-mid perivascular and interstitial infiltrate consisting of neutrophils and eosinophils, accompanied by leukocytoclasia and extravasated erythrocytes, and fibrin deposition in the vessel walls (Fig 2). The clinical history of tender, edematous plaques lasting several days, in conjunction with the observed histopathologic findings, confirmed a diagnosis of urticarial vasculitis. Serum complement levels (including C3, C4, and C1q), complete blood count, and comprehensive metabolic panel were within normal limits. Antinuclear antibodies were negative. Eight days before dermatology consult, a 40 mg prednisone taper was initiated for the rash and atezolizumab was held due to the rash. The rash resolved the day after the consult. There was no residual postinflammatory hyperpigmentation. Atezolizumab was never resumed due to suspected ICI-induced pneumonitis which developed 7 weeks after rash onset. A computed tomography scan 3 months after atezolizumab initiation demonstrated partial tumor response.

## Discussion

Urticarial vasculitis is believed to be a type III hypersensitivity reaction mediated by immune complex deposition of C3 and immunoglobulins in the postcapillary venules.^6^ Antibody-triggered release of C3a and C5a leads causes chemotaxis of neutrophils and eosinophils. Concurrent activation of mast cells further contributes to the generation of angioedema and urticarial lesions characteristic of urticarial vasculitis. The underlying pathogenesis is reflected in histopathologic findings, which often include damaged dermal blood vessels, neutrophil karyorrhexis, extravasation of erythrocytes into the dermis, and an inflammatory infiltrate composed of neutrophils, eosinophils, and lymphocytes.^5^

The severity of urticarial vasculitis can vary, ranging from mild skin symptoms to systemic involvement requiring immunosuppressive therapy. The degree of complement consumption dictates the disease severity.^7^ In skin-limited cases, complement levels typically remain within the normal range. Contrarily, hypocomplementemic urticarial vasculitis is associated with a poorer prognosis and may present with various systemic symptoms, such as renal, lung, and gastrointestinal involvement. Pulmonary involvement in systemic cases is the leading cause of death for urticarial vasculitis; thus pulmonary function testing is indicated for symptomatic patients.^5^ Systemic lupus erythematosus can cooccur with hypocomplementemic urticarial vasculitis, and there is a high degree of symptom overlap between the 2 conditions. Therefore, when evaluating a patient for urticarial vasculitis, the initial workup should include complement levels and antinuclear antibodies, in addition to a complete blood count and basic chemistries.^5^

The presented case suggests an association between the PD-L1 inhibitor atezolizumab and the development of urticarial vasculitis in a patient with metastatic small cell lung cancer. The temporal relationship between the initiation of atezolizumab therapy and the onset of cutaneous symptoms strongly suggests a drug-induced etiology. Moreover, cirAE development is associated with favorable ICI response,^8^ as seen in this case. Although urticarial vasculitis secondary to ICI therapy is, to our knowledge, not a previously reported phenomenon, reports have characterized the development of other types of vasculitis in response to ICI treatment.^9^ Notably, the PD-L1 pathway has been implicated in the development of vasculitis, particularly giant cell arteritis.^9^ In a transcriptomic analysis of giant cell arteritis, arterial biopsies demonstrated low PD-L1 expression, suggesting that inhibition of programmed death-1 and PD-L1 leads to cytokine release and loss of immune privilege in the vessel wall.^9^^,^^10^ In the presented case, it is feasible that inhibition of PD-L1 by atezolizumab contributed to the activation of the aforementioned inflammatory mediators, leading to the development of an ICI-induced urticarial vasculitis.

There are no treatment guidelines for urticarial vasculitis, and randomized controlled trials evaluating the efficacy of available therapies are lacking. However, several therapies have been utilized with varying levels of success, including: doxycycline, dapsone, colchicine, corticosteroids, omalizumab, rituximab, and anakinra.^5^^,^^7^ As urticarial vasculitis can be associated with medications, cancers, or infections, withdrawal of the offending agent, cancer remission, or treatment of the underlying infection can effectively improve urticarial vasculitis symptoms.^7^ Early diagnosis and management of cirAEs is crucial to limit interruptions or discontinuation of life-saving cancer therapy.

In reporting this case, we hope to contribute to the literature regarding the spectrum of cirAEs. Dermatologists should be aware of the various cirAE presentations in order to guide workup and management. Further research should elucidate the underlying mechanisms and optimize treatment strategies for this cutaneous manifestation.

## Conflicts of interest

None disclosed.
